# Multicomponent synthesis and anticonvulsant activity of monocyclic 2,6-diketopiperazine derivatives

**DOI:** 10.1007/s00044-013-0800-4

**Published:** 2013-10-01

**Authors:** Maciej Dawidowski, Jadwiga Turło

**Affiliations:** Department of Drug Technology and Pharmaceutical Biotechnology, Medical University of Warsaw, Banacha 1 Str., 02-097 Warsaw, Poland

**Keywords:** Multicomponent reactions, Ugi reaction, Anticonvulsant activity, 2,6-Diketopiperazine, 2,6-Piperazinedione

## Abstract

In this study, a series of diastereomerically pure monocyclic 2,6-diketopiperazine (2,6-DKP) derivatives were synthesized. The key synthetic step involved a multicomponent Ugi five-center, four-component reaction which was used to generate the convertible *tert*-butylamidoesters with both good yields and high diastereoselectivity toward the desired bioactive (*S*,*S*) absolute configuration. In subsequent steps, selective *tert*butyl cleavage by use of BF_3_·CH_3_COOH and base-induced intramolecular cyclocondensation gave the final 2,6-DKP derivatives. The relative stereochemistry of the target molecules was confirmed by ^1^H NMR experiments. The compounds obtained were submitted to in vivo screening in animal models of epilepsy. Some of them displayed good activity in maximal electroshock seizure and 6 Hz tests.

## Introduction

Epilepsy is a major neurological disorder characterized by recurrent, spontaneous seizures. It affects approx. 50 million people (~1 % of the world’s population). Currently, the main treatment for epilepsy is the chronic administration of anticonvulsant drugs (AEDs). Although more than 30 AEDs are available, they provide satisfactory seizure control in only 60 % of patients. Additionally, major concerns of pharmacotherapy of epilepsy include high incidence of severe side effects and drug–drug interactions resulting from enzyme induction. Therefore, there is substantial need for new, more effective and safer AEDs (Kwan and Brodie, 2000; Brodie, 2001). Another essential challenge for epilepsy research is to develop therapeutics that would not only symptomatically suppress seizures, but would also inhibit or reverse progression of the sickness (the so-called “disease modifying” drugs; Perucca *et al*., 2007; Bialer and White, 2010).

Presently, the compounds at different stages of development belong to various chemical classes and display diverse, often unknown mechanisms of action (Bialer *et al*., 2013). Most of these agents have been identified initially through in vivo screening in animal models of epilepsy rather than by a mechanistic approach. Although the animal models utilized for screening are associated with certain endpoints, it is generally accepted that they offer a good starting point in the early discovery of new AED candidates (Löscher and Schmidt, 1994; Malawska, 2005; Rogawski, 2006; Smith *et al.*, 2007; Bialer and White, 2010; Banerjee and Sharma, 2012; Mishra and Ganguly, 2012).

**Fig. 1:**
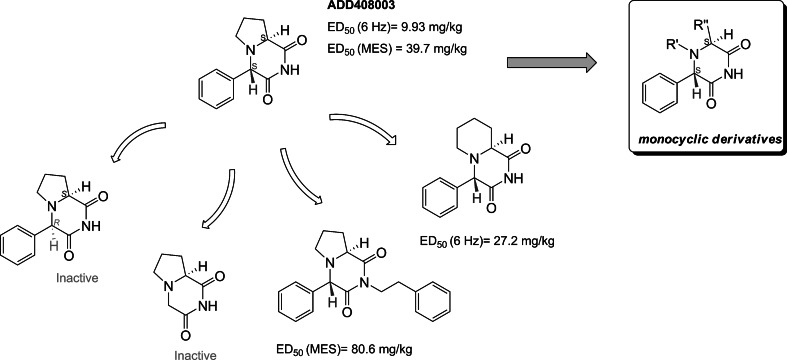
Preliminary SAR of anticonvulsant 2,6-DKPs and proposed structural modifications

Recently, we have reported that chiral, bicyclic 2,6-diketopiperazines (2,6-DKPs) derived from cyclic amino acids display a broad anticonvulsant activity in various animal models of epilepsy (Dawidowski *et al.*, 2011, 2012a). Among the newly developed agents, compound ADD408003 exhibited a broad spectrum of seizure-suppressing activity. A preliminary structure–activity relationship (SAR) study of close analogs revealed that several factors are responsible for the anticonvulsant activity (Fig. 1): the (*S*,*S*) absolute configuration on the stereogenic centers, the presence of imide moiety and the benzene ring adjacent to 2,6-DKP core. Further, neither substitution of the imide nitrogen of ADD408003 with different alkyl and arylalkyl moieties nor expansion of the fused pyrrole chain markedly influenced the antiseizure activity.

These findings led us to ask whether the related monocyclic 2,6-DKPs, derived from non-polar l-amino acids other than l-proline or l-homoproline display comparable anticonvulsant activity. The designed compounds fulfill all requirements determined on the basis of the preliminary SAR analysis, i.e., proper stereochemistry, the presence of imide moiety and benzene ring attached to 2,6-DKP scaffold. Further, due to the absence of the fused pyrrolidine or piperidine rings, these agents are less sterically constrained, which might allow for a better fit to the putative receptor(s).

## Results and discussion

### Chemistry

**Scheme 1:**
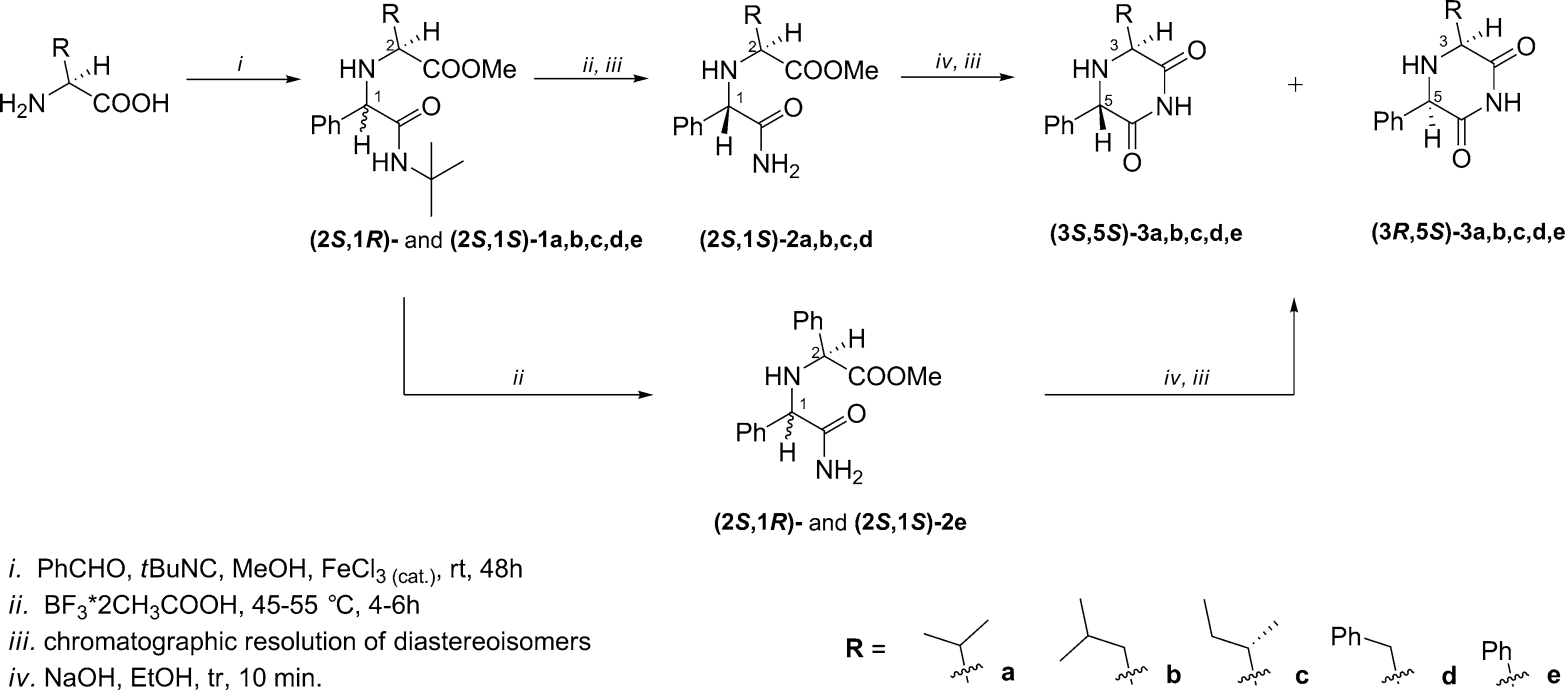
Synthesis of enantiopure 2,6-DKP derivatives **3a**–**e**

The target enantiopure, monocyclic 2,6-DKP derivatives **3a–e** were synthesized according to the reaction sequences depicted in Scheme 1.

In the first step, the Ugi five-center four-component reaction (U-5C-4CR; Demharter *et al.*, 1996) of the appropriate non-polar amino acid (l-valine, l-leucine, l-isoleucine, l-phenylalanine, l-phenylglycine), benzaldehyde, *tert*-butyl isocyanide, and methanol in the presence of a catalytic amount of iron(III) chloride gave the *tert*-butylamidoesters **1a–e** with chemical yields ranging from 41 to 75 %. The reaction proceeded with the formation of the new stereocenter and in all cases, the major diastereomer was **(2** ***S*** **,1** ***S*** **)-1**, as judged by the ^1^H NMR analyses of the crude post-reaction mixtures. In general, the degree of diastereoinduction depended on the steric bulkiness of the side chain of the substrate amino acid. The highest diastereomeric ratios were measured for l-valine and l-isoleucine derivatives **1a** (*d*_r_ = 7.3/1) and **1c** (*d*_r_ = 9.0/1), respectively, bearing branched alkyl chains directly adjacent to the position C-2, located close to the newly formed stereocenter. The U-5C-4CR adducts of l-leucine and l-phenylalanine **1b** and **d**, respectively, were formed with a slightly lower diastereoinduction (*d*_r_ ≈ 5/1 for each). This could be attributed to the lower steric hindrance of a methylene group adjacent to the carbon C-2. A surprisingly small degree of diastereoselectivity was found for the l-phenylglycine derivative **1e** (*d*_r_ = 1.5/1), having a bulky phenyl substituent in the position C-2. The possible explanation for this unexpected observation is the stabilization of the six-membered cyclic Ugi intermediate (Demharter *et al.*, 1996) leading to **(2** ***S*** **,1** ***R*** **)-1e** by a pi–pi interaction of the two phenyl rings occupying axial positions.

Attempts to quantitatively separate the diastereoisomers of **1a–e** by column chromatography or fractional recrystallization failed. Therefore, the obtained diastereomeric mixtures were used in the subsequent amide N-de*tert*butylation. Reaction of **(2** ***S*** **,1** ***S*** **)**/**(2** ***S*** **,1** ***R*** **)-1a–e** with BF_3_·CH_3_COOH at 45–55 °C provided amidoesters **2a–e** with the yields range from 55 to 83 %. With the exception of **2e**, all diastereomeric mixtures could be efficiently resolved by column chromatography.

In the last step, compounds **(2** ***S*** **,1** ***S*** **)-2a–d** were subjected to base-induced intramolecular cyclization. The reaction was accompanied by a notable degree of epimerization at stereogenic centers C-5 of the products **3**. Nevertheless, in all cases, the unwanted **(3** ***S*** **,5** ***R*** **)** isomers could be separated by means of column chromatography (compounds **(3** ***S*** **,5** ***R*** **)-3a**, **c**, **d**) or recrystallization (compound **(3** ***S*** **,5** ***R*** **)-3b**). Intramolecular cyclization of 1.4/1 diastereomeric mixture of **(2** ***S*** **,1** ***S*** **)**/**(2** ***S*** **,1** ***R*** **)-2e** gave **(3** ***S*** **,5** ***S*** **)-3e** and **(3** ***S*** **,5** ***R*** **)-3e** (a *meso* compound) in equal proportion. The isomers were efficiently separated by column chromatography.

**Fig. 2:**
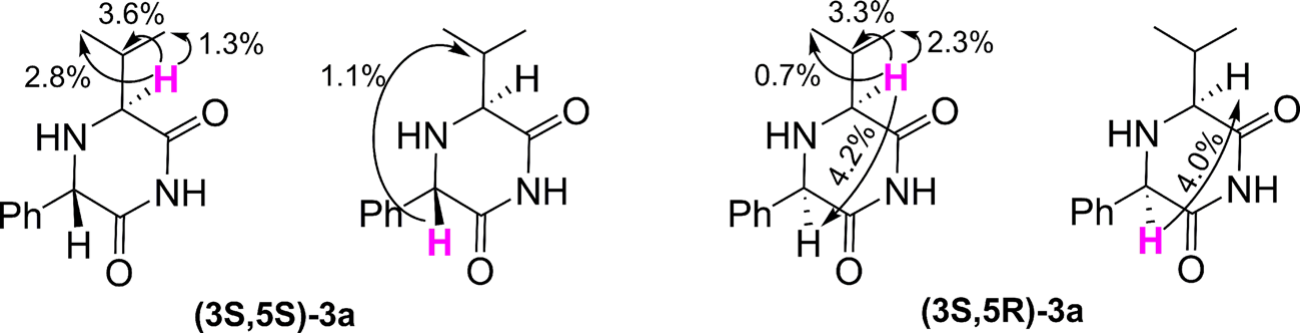
Selected nOe correlations in **(3** ***S*** **,5** ***S*** **)-3a** and **(3** ***S*** **,5** ***R*** **)-3a**

Relative stereochemistry of the respective diastereoisomers of 2,6-DKPs **3** was confirmed with nuclear Overhauser effect (nOe) ^1^H NMR experiments (Fig. 2) performed for **(3** ***S*** **,5** ***S*** **)** and **(3** ***S*** **,5** ***R*** **)-3a**. Contrary to **(3** ***S*** **,5** ***R*** **)-3a**, the interatomic distance between protons H-3 and H-5 in **(3** ***S*** **,5** ***S*** **)-3a** should exclude any considerable nOe effect. Indeed, the irradiation of the H-3 resonance in **(3** ***S*** **,5** ***R*** **)-3a** resulted in a remarkable enhancement of the H-5 signal (4.2 %), whereas no increase in intensity for the proton H-5 was observed in the analogous experiment performed for **(3** ***S*** **,5** ***S*** **)-3a**. The reverse order of irradiation, consisting in saturation of H-5, led to the enhancement of H-3 resonance in **(3** ***S*** **,5** ***R*** **)-3a** (4.0 %), but not in **(3** ***S*** **,5** ***S*** **)-3a**.

The difference in the chemical shifts of the protons H-3 and H-5 adjacent to the stereogenic carbon atoms was another ^1^H NMR spectroscopic feature useful for distinguishing between the respective diastereoisomers of **3**. With the exception of **3e**, the signals corresponding to H-5 in **(3** ***S*** **,5** ***S*** **)-3** were shifted downfield (Δ*δ* = 0.26–0.38 ppm [parts per million]) compared to those of **(3** ***S*** **,5** ***R*** **)-3**. This observation was in agreement with the pseudoequatorial arrangement of these protons with respect to the 2,6-DKP ring in **(3** ***S*** **,5** ***S*** **)-3**, and their pseudoaxial position in **(3** ***S*** **,5** ***R*** **)-3**. On the contrary, resonances of the H-3 protons in all **(3** ***S*** **,5** ***S*** **)-3** isomers were shifted upfield (Δ*δ* = 0.14–0.32 ppm). Although these protons in both respective diastereoisomers occupy the same pseudoaxial positions, the slightly stronger shielding in **(3** ***S*** **,5** ***R*** **)-3** could be attributed to the anisotropic effect of the phenyl ring present in the spatial vicinity.

**Scheme 2:**
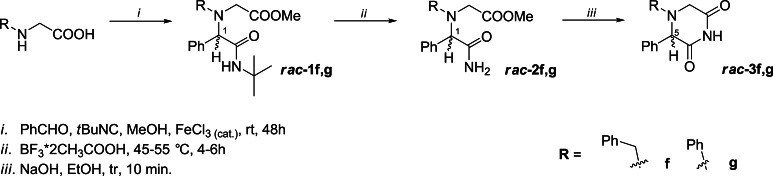
Synthesis of racemic 2,6-DKP derivatives **3f**, **g**

The racemic 2,6-DKPs **3f**, **g** were synthesized from the corresponding *N*-substituted glycines in a similar manner, according to the reaction sequences depicted in Scheme 2. Notably, the chemical yields of racemic U-5C-4CR products **1f** and **g** (18 and 24 %, respectively) were significantly lower than those observed for *N*-unsubstituted adducts **1a–e**.

### Anticonvulsant screening

Compounds **3a–f** were evaluated in the in vivo animal models of epilepsy within the Anticonvulsant Screening Program (ASP) of The National Institute of Neurological Disorders and Stroke (NINDS), at The National Institutes of Health according to well-established protocols described in the “Experimental” section of this article. The compounds were screened using maximal electroshock seizure (MES) and subcutaneous metrazole (scMET) tests (White *et al.*, 2002). The first of these tests uses an electrical stimulus to induce generalized tonic–clonic seizures and is capable of identifying compounds that prevent the spread of seizure. The latter employs chemically induced seizures to recognize agents that raise the seizure threshold. The most promising compounds were subjected to an evaluation of anticonvulsant activity using a minimal clonic seizure (6 Hz) test (Brown *et al.*, 1953; Barton *et al.*, 2001; Kaminski *et al.*, 2004), which is regarded as a preliminary model of pharmacoresistant limbic seizures. Additionally, a standardized *rotorod* test for neurological toxicity (TOX; Dunham and Miya, 1957) was performed for each compound. The results are summarized in Tables 1 and 2.

**Table 1:** Anticonvulsant activity and neurotoxicity of compounds in the MES and scMET models following intraperitoneal (ip) administration in mice

Compounds	Dose (mg/kg)	MES^a^		scMET^b^		TOX^c^		*C*log*P* ^d^
		0.5 h	4.0 h^e^	0.5 h	4.0 h	0.5 h	4.0 h^e^	
**(3** ***S*** **,5** ***S*** **)-3a**	30	0/1	0/1	0/1	0/1	0/4	0/2	0.80
	100	**2/3**	0/3	0/1	0/1	0/8	0/4	
	300	**1/1**	0/1	0/1	0/1	**4/4** ^f, g^	0/2	
**(3** ***S*** **,5** ***R*** **)-3a**	30	0/1	0/1	0/1	0/1	0/4	0/2	0.80
	100	0/3^h^	0/3	**1/5** ^i^	0/1	0/8	0/4	
	300	**1/1**	0/1	0/1	0/1	**2/4**	0/2	
**(3** ***S*** **,5** ***S*** **)-3b**	30	0/1	0/1	0/1	0/1	0/4	0/2	1.19
	100	0/3	0/3	0/1	0/1	0/8	0/4	
	300	**1/1**	0/1	0/1	0/1	**3/4** ^f^	0/2	
**(3** ***S*** **,5** ***S*** **)-3c**	30	0/1	0/1	0/1	0/1	0/4	0/2	1.19
	100	0/3	0/3	0/1	0/1	0/8	0/4	
	300	0/1	0/1	0/1	0/1	**2/4**	0/2	
**(3** ***S*** **,5** ***S*** **)-3d**	30	0/1	0/1	0/1	0/1	0/4	0/2	1.61
	100	0/3	0/3	0/1	0/1	0/8	0/4	
	300	**1/1**	0/1	0/1	0/1	0/4	0/2	
**(3** ***S*** **,5** ***S*** **)-3e**	30	0/4	0/4	–	–	0/8	0/8	2.12
	100	**2/4**	**1/4**	–	–	0/8	0/8	
	300	**4/4**	**4/4**	–	–	**2/8**	**1/8**	
**(3** ***S*** **,5** ***R*** **)-3e**	30	0/4	0/4	–	–	0/8	0/8	2.12
	100	0/4	0/4	–	–	0/8	0/8	
	300	**1/4**	0/4	–	–	0/8	0/8	
***rac*** **-3f**	30	0/4	0/4	–	–	0/8	0/8	2.29
	100	0/4	0/4	–	–	0/8	0/8	
	300	0/4	**3/4**	–	–	0/8	0/8	
***rac*** **-3g**	30	0/1	0/1	0/1	0/1	0/4	0/2	2.12
	100	0/3	0/3	0/1	0/1	0/8	0/4	
	300	0/1	0/1	0/1	0/1	0/4	0/2	

Ratios where at least one animal was protected or displayed neurotoxicity have been highlighted in bold to enhance data readability and interpretation

^a^Maximal electroshock test (number of animals protected/number of animals tested)

^b^Subcutaneous metrazole test (number of animals protected/number of animals tested)

^c^Neurotoxicity test (number of animals exhibiting neurological toxicity/number of animals tested)

^d^Theoretical log*P* value calculated by a logarithm included in HyperChem 7.5 package

^e^Compounds **(3** ***S*** **,5** ***S*** **)-3e**, **(3** ***S*** **,5** ***R*** **)-3e** and ***rac*** **-3f** were tested at 2.0 h post administration

^f^Unable to grasp rotorod

^g^Loss of righting reflex

^h^Active also in 1/3 at 0.25 h post administration

^i^Myoclonic jerks

**Table 2:** Anticonvulsant activity and neurotoxicity of compounds in the 6 Hz model following intraperitoneal (ip) administration in mice

Compounds	Test^a^	0.25 h	0.5 h	1.0 h	2.0 h	4.0 h
**(3** ***S*** **,5** ***S*** **)-3a**	6 Hz^b^	**2/4**	**1/4**	0/4	0/4	0/4
	TOX^c^	0/4	0/4	0/4	0/4	0/4
**(3** ***S*** **,5** ***S*** **)-3e**	6 Hz	–	0/4	–	0/4	–
	TOX	–	0/8	–	0/8	–

Ratios where at least one animal was protected or displayed neurotoxicity have been highlighted in bold to enhance data readability and interpretation

^a^At dose 100 mg/kg

^b^6 Hz test, 32 mA (number of animals protected/number of animals tested)

^c^Neurotoxicity test (number of animals exhibiting neurological toxicity/number of animals tested)

As shown in Table 1, compounds **3a**, **b**, **d–f** exhibited weak to good anticonvulsant activities in the MES model in mice. **(3** ***S*** **,5** ***S*** **)-3a** and **(3** ***S*** **,5** ***S*** **)-3e** were most potent, displaying a notable seizure protection in 2/3 and 2/4 of tested animals, respectively, at the dose of 100 mg/kg, after 0.5 h postadministration. No neurotoxicity was detected at the same dose. **(3** ***S*** **,5** ***S*** **)-3e** also proved effective also at the later time points (1/4 and 4/4 at 100 and 300 mg/kg, respectively, at 2.0 h). None of the compounds investigated were significantly active in the scMET model.

According to our previous reports on bicyclic 2,6-DKP derivatives, the (*R*,*S*) isomers were completely devoid of any in vivo pharmacological activity (Dawidowski *et al.*, 2011, 2012a). In contrast, the monocyclic derivatives **(3** ***R*** **,5** ***S*** **)-3a** and **(3** ***R*** **,5** ***S*** **)-3e**, displayed a weak, yet noticeable activity in the MES test (1/1 and 1/4 at 300 mg, respectively, at 0.5 h). This could mean that the greater flexibility due to the lack of fused alkyl rings allows for a better fit in the putative binding site within the CNS. Nevertheless, the (*S*,*S*) isomers again proved more active.

In general, the most significant levels of seizure protection were observed for derivatives bearing alkyl or aryl substituents at carbon C-5. Among the compounds with alkyl groups, the l-valine derivative with isopropyl side chain **(3** ***S*** **,5** ***S*** **)-3a** was most potent in the MES test. High levels of seizure protection was also observed for symmetrical **(3** ***S*** **,5** ***S*** **)-3e** having two benzene rings with a proper stereochemistry with respect to the 2,6-DKP core. Importantly, the anticonvulsant activity of the investigated molecules was not dependant on their log*P* values.

Derivatives **(3** ***S*** **,5** ***S*** **)-3a** and **(3** ***S*** **,5** ***S*** **)-3e** were further assessed for their potential efficacy against pharmacoresistant epilepsy using the 6 Hz screen. The results are summarized in Table 2. Notable activity was detected for the first compound (2/4 and 1/4, at 0.25 and 0.5 h, respectively, at 100 mg/kg), while the latter was inactive.

## Conclusions

We have synthesized a series of novel diastereomerically pure, monocyclic 2,6-DKP derivatives by use of diastereoselective synthetic sequence using the U-5C-4CR multicomponent reaction as the key step. The compounds displayed weak to good anticonvulsant activities in the MES model, while none of them were active in scMET screen. The most promising compound **(3** ***S*** **,5** ***S*** **)-3a** exhibited notable action in the 6 Hz test. Contrary to the recently reported activity of bicyclic 2,6-DKPs, the activity of monocyclic derivatives seemed to be less stereochemistry-dependent. We conclude that this is due to increased conformational flexibility. Although the seizure-suppressing potency of the newly synthesized agents was generally weaker relative to bicyclic 2,6-DKPs, they possess secondary amino groups that provide additional points of diversification for further SAR studies.

## Experimental

### Chemistry

Melting points were determined on an Electrothermal 9100 apparatus in open capillary tubes and were uncorrected. The IR spectra (thin film on KBr pellets) were recorded on a Shimadzu FTIR-8300 instrument. The NMR spectra were obtained on a Varian Inova 500 MHz spectrometer. Chemical shifts (*δ*) were expressed in ppm relative to tetramethylsilane or solvent used as the internal reference. The following abbreviations were used to describe the peak patterns: s (singlet), d (doublet), t (triplet), q (quartet), qt (quintet), sp (septet), m (multiplet), p (pseudo-), and b (broad-). Coupling constants (*J*) were in hertz (Hz). The electrospray ionization high-resolution mass spectra (ESI-HRMS) were recorded on a LCT TOF (Micromass) instrument. Optical rotations were measured with a Perkin-Elmer 241 polarimeter at 20 °C, using a sodium lamp (589 nm). Thin-layer chromatography (TLC) was run on Merck Silica gel-60 F_254_ plates. The spots were visualized by ultraviolet light (254 nm) or iodine vapors. Flash column chromatography (FC) was carried out on Merck Silica gel 60 (particle size 0.040–0.063 mm). Solvents were dried and purified by standard methods. Petroleum ether (PE) referred to the fraction boiling at 40–60 °C. All reagents were purchased from commercial sources and used as received. Unless otherwise stated, the chemical yields were calculated for pure (*d*_r_ ≥95/5) compounds. Compound ***rac*** **-3g** was synthesized as described previously (Dawidowski *et al.*, 2012b).

### Synthesis of compounds **1** by U-5C-4CR condensation

Iron(III) chloride (5 mol.%) and *tert*-butyl isocyanide (1.0 equiv.) were added to a stirred suspension of appropriate α-amino acid (1.2 equiv.) and benzaldehyde (1.0 equiv.) in MeOH (100 mL). The mixture was stirred at RT for 48 h and the volatiles were removed under reduced pressure. The resulting crude products were purified FC.

#### Methyl (2*S*,1*S*)- and (2*S*,1*R*)-2-(2-(*tert*-butylamino)-2-oxo-1-phenylethylamino)-3-methylbutanoate **(2*S*,1*S*)-1a** and **(2*S*,1*R*)-1a**

From l-valine (2.36 g, 20.16 mmol), benzaldehyde (16.80 mmol, 1.71 mL) and *tert*-butyl isocyanide (2.00 mL, 16.80 mmol); FC (gradient: PE/AcOEt 6:1–3:1): yield 4.04 g (75 %) of chromatographically inseparable diastereomeric mixture (*d*_r_ = 7.3/1, ^1^H NMR). Analytical sample of **(2** ***S*** **,1** ***S*** **)-1a** was obtained by recrystallization from PE/Et_2_O 10:1. **(2** ***S*** **,1** ***S*** **)-1a**: white wax; mp 37–38 °C; [*α*]_D_ = −97.2 (*c* 1, CHCl_3_); IR (KBr): 729, 764, 1200, 1454, 1516, 1678, 1736, 2874, 2962, 3333; TLC (PE/AcOEt 3:1): *R*_f_ = 0.43; ^1^H NMR (CDCl_3_, 500 MHz): *δ* 0.89 (d, ^3^ *J* = 6.5, 3H, C*H*_3_), 0.93 (d, ^3^ *J* = 6.5, 3H, ${\rm C}H_{3}^{'}$), 1.29 (s, 9H, C(C*H*_3_)_3_), 1.96 (m, ^3^ *J* = 6.5, 1H, C*H*), 2.34 (bs, 1H, N*H*), 2.87 (bpd, ^3^ *J* = 5.0, 1H, H-2), 3.71 (s, 3H, OC*H*_3_), 4.08 (s, 1H, H-1), 6.37 (bs, 1H, CON*H*), 7.28–7.36 (m, 5H, H–Ar); ^13^C NMR (CDCl_3_, 125 MHz): *δ* 18.4 (*C*H_3_), 19.2 ($C{\text{H}}_{3}^{'}$), 28.6 (C(*C*H_3_)_3_), 31.4 (*C*H), 50.8 (*C*(CH_3_)_3_), 51.5 (O*C*H_3_), 64.6 (C-2), 66.6 (C-1), 127.9 (C-2′, C-6′), 128.2 (C-4′), 128.8 (C-3′, C-5′), 138.8 (C-1′), 170.9 (*C*ONH), 174.7 (*C*OOCH_3_); HRMS (ESI) calcd for C_18_H_28_N_2_O_3_Na: 343.1998 (M+Na)^+^ found 343.1958. **(2** ***S*** **,1** ***R*** **)-1a**: ^1^H NMR (from diastereomeric mixture, CDCl_3_, 500 MHz): 0.95 (d, ^3^ *J* = 6.5, 3H, C*H*_3_), 1.06 (d, ^3^ *J* = 6.5, 3H, ${\rm C}H_{3}^{'}$), 1.39 (s, 9H, C(C*H*_3_)_3_), 2.02 (m, ^3^ *J* = 6.5, 1H, C*H*), 2.34 (bs, 1H, N*H*), 3.09 (m, 1H, H-2), 3.73 (s, 3H, OC*H*_3_), 3.92 (s, 1H, H-1), 6.37 (bs, 1H, CON*H*), the remaining signals overlap with the signals of **(2** ***S*** **,1** ***S*** **)-1a**; ^13^C NMR (from diastereomeric mixture, CDCl_3_, 125 MHz): *δ* 18.0 (*C*H_3_), 19.6 ($C{\text{H}}_{3}^{'}$), 28.8 (C(*C*H_3_)_3_), 31.5 (*C*H), 50.7 (*C*(CH_3_)_3_), 51.8 (O*C*H_3_), 66.3 (C-1), 67.0 (C-2), 127.3 (C-2′, C-6′), 128.3 (C-4′), 128.8 (C-3′, C-5′), 139.6 (C-1′), 170.9 (*C*ONH), 175.0 (*C*OOCH_3_).

#### Methyl (2*S*,1*S*)- and (2*S*,1*R*)-2-(2-(*tert*-butylamino)-2-oxo-1-phenylethylamino)-4-methylpentanoate **(2*S*,1*S*)-1b** and **(2*S*,1*R*)-1b**

From l-leucine (2.64 g, 20.16 mmol), benzaldehyde (16.80 mmol, 1.71 mL) and *tert*-butyl isocyanide (2.00 mL, 16.80 mmol); FC (gradient: PE/AcOEt 9:1–4:1): yield 3.58 g (64 %) of diastereomeric mixture (*d*_r_ = 5.3/1, ^1^H NMR). Colorless oil; IR (KBr): 700, 733, 1155, 1200, 1227, 1454, 1516, 1680, 1738, 2870, 2959, 3331; TLC (PE/AcOEt 3:1): *R*_f_ = 0.35 (major isomer) and 0.38 (minor isomer); ^1^H NMR (from diastereomeric mixture, CDCl_3_, 500 MHz): **(2** ***S*** **,1** ***S*** **)-1b** (major isomer): *δ* 0.77 (d, ^3^ *J* = 6.5, 3H, C*H*_3_), 0.87 (d, ^3^ *J* = 6.5, 3H, ${\rm C}H_{3}^{'}$), 1.31 (s, 9H, C(C*H*_3_)_3_), 1.58 (m, 2H, C*H*_2_), 1.71 (m, ^3^ *J* = 6.5, 1H, C*H*), 2.26 (bs, 1H, N*H*), 3.11 (pt, ^3^ *J* = 7.5, 1H, H-2), 3.70 (s, 3H, OC*H*_3_), 4.11 (s, 1H, H-1), 6.49 (bs, 1H, CON*H*), 7.28–7.37 (m, 5H, H–Ar); **(2** ***S*** **,1** ***R*** **)-1b** (minor isomer): *δ* 0.96 (d, ^3^ *J* = 6.5, 3H, C*H*_3_), 0.99 (d, ^3^ *J* = 6.5, 3H, ${\rm C}H_{3}^{'}$), 1.38 (s, 9H, C(C*H*_3_)_3_), 1.86 (m, ^3^ *J* = 6.5, 1H, C*H*), 3.32 (dd, ^3^ *J*_1_ = 9.0, ^3^ *J*_2_ = 5.0, 1H, H-2), 3.72 (s, 3H, OC*H*_3_), 3.95 (s, 1H, H-1), the remaining signals overlap with the signals of **(2** ***S*** **,1** ***S*** **)-1b**; ^13^C NMR (from diastereomeric mixture, CDCl_3_, 125 MHz): **(2** ***S*** **,1** ***S*** **)-1b** (major isomer): *δ* 22.0 (*C*H_3_), 22.8 ($C{\text{H}}_{3}^{'}$), 24.8 (*C*H), 28.6 (C(*C*H_3_)_3_), 42.5 (*C*H_2_), 50.9 (*C*(CH_3_)_3_), 51.2 (O*C*H_3_), 57.5 (C-2), 66.4 (C-1), 127.8 (C-2′, C-6′), 128.2 (C-4′), 128.9 (C-3′, C-5′), 139.0 (C-1′), 170.8 (*C*ONH), 175.4 (*C*OOCH_3_); **(2** ***S*** **,1** ***R*** **)-1b** (minor isomer): *δ* 22.2 (*C*H_3_), 23.2 ($C{\text{H}}_{3}^{'}$), 24.9 (*C*H), 28.7 (C(*C*H_3_)_3_), 43.4 (*C*H_2_), 50.7 (*C*(CH_3_)_3_), 52.0 (O*C*H_3_), 59.0 (C-2), 66.9 (C-1), 127.2 (C-2′, C-6′), 128.1 (C-4′), 128.8 (C-3′, C-5′), 139.9 (C-1′), 170.9 (*C*ONH), 175.9 (*C*OOCH_3_); HRMS (ESI) calcd for C_18_H_28_N_2_O_3_Na: 357.2154 (M+Na)^+^ found 357.2171.

#### Methyl (2*S*,1*S*,3*S*)- and (2*S*,1*R*,3*S*)-2-(2-(*tert*-butylamino)-2-oxo-1-phenylethylamino)-3-methylpentanoate **(2*S*,1*S*,3*S*)-1c** and **(2*S*,1*R*,3*S*)-1c**

From l-isoleucine (2.64 g, 20.16 mmol), benzaldehyde (16.80 mmol, 1.71 mL) and *tert*-butyl isocyanide (2.00 mL, 16.80 mmol); FC (gradient: PE/AcOEt 9:1–4:1): yield 3.97 g (71 %) of chromatographically inseparable diastereomeric mixture (*d*_r_ = 9.0/1, ^1^H NMR). Colorless oil; IR (KBr): 700, 741, 1148, 1200, 1225, 1265, 1454, 1516, 1678, 1736, 2876, 2930, 2964, 3329; TLC (PE/AcOEt 3:1): *R*_f_ = 0.35; ^1^H NMR (from diastereomeric mixture, CDCl_3_, 500 MHz): **(2** ***S*** **,1** ***S*** **,3** ***S*** **)-1c** (major isomer): *δ* 0.83 (t, ^3^ *J* = 7.5, 3H, CH_2_C*H*_3_), 0.85 (d, ^3^ *J* = 7.0, 3H, C*H*_3_), 1.16 (m, 1H, C*H*_2_), 1.30 (s, 9H, C(C*H*_3_)_3_), 1.51 (m, 1H, ${\rm C}H_{2}^{'}$), 1.72 (m, 1H, C*H*), 2.35 (bs, 1H, N*H*), 2.94 (d, ^3^ *J* = 6.0, 1H, H-2), 3.71 (s, 3H, OC*H*_3_), 4.07 (s, 1H, H-1), 6.37 (bs, 1H, CON*H*), 7.27–7.34 (m, 5H, H–Ar); **(2** ***S*** **,1** ***R*** **,3** ***S*** **)-1c** (minor isomer): *δ* 0.92 (t, ^3^ *J* = 7.5, 3H, CH_2_C*H*_3_), 1.00 (d, ^3^ *J* = 7.0, 3H, C*H*_3_), 1.39 (s, 9H, C(C*H*_3_)_3_), 3.17 (d, ^3^ *J* = 5.5, 1H, H-2), 3.72 (s, 3H, OC*H*_3_), 3.93 (s, 1H, H-1), 5.30 (bs, 1H, CON*H*), the remaining signals overlap with the signals of **(2** ***S*** **,1** ***S*** **,3** ***S*** **)-1c**; ^13^C NMR (from diastereomeric mixture, CDCl_3_, 125 MHz): **(2** ***S*** **,1** ***S*** **,3** ***S*** **)-1c** (major isomer): *δ* 11.3, 15.6 (*C*H_3_, $C{\text{H}}_{3}^{'}$), 25.3 (*C*H_2_), 28.6 (C(*C*H_3_)_3_), 38.0 (*C*H), 50.9 (*C*(CH_3_)_3_), 51.5 (O*C*H_3_), 63.5 (C-2), 66.6 (C-1), 127.9 (C-2′, C-6′), 128.2 (C-4′), 128.8 (C-3′, C-5′), 138.8 (C-1′), 170.9 (*C*ONH), 174.7 (*C*OOCH_3_); **(2** ***S*** **,1** ***R*** **,3** ***S*** **)-1c** (minor isomer): *δ* 11.7, 16.4 (*C*H_3_, $C{\text{H}}_{3}^{'}$), 25.0 (*C*H_2_), 28.8 (C(*C*H_3_)_3_), 38.5 (*C*H), 50.7 (*C*(CH_3_)_3_), 51.7 (O*C*H_3_), 65.3 (C-2), 67.1 (C-1), 127.2 (C-2′, C-6′), 128.0 (C-4′), 128.8 (C-3′, C-5′), 139.6 (C-1′), 171.0 (*C*ONH), 174.7 (*C*OOCH_3_); HRMS (ESI) calcd for C_18_H_28_N_2_O_3_Na: 357.2154 (M+Na)^+^ found 357.2148.

#### Methyl (2*S*,1*S*)- and (2*S*,1*R*)-2-(2-(*tert*-butylamino)-2-oxo-1-phenylethylamino)-3-phenylpropanoate **(2*S*,1*S*)-1d** and **(2*S*,1*R*)-1d**

From l-phenylalanine (3.33 g, 20.16 mmol), benzaldehyde (16.80 mmol, 1.71 mL) and *tert*-butyl isocyanide (2.00 mL, 16.80 mmol); FC (gradient: PE/AcOEt 9:1–2:1): yield 3.23 g (52 %) of diastereomeric mixture (*d*_r_ = 5.1/1, ^1^H NMR). Pale-yellow oil; IR (KBr): 700, 754, 1223, 1454, 1516, 1680, 1738, 2872, 2966, 3326; TLC (PE/AcOEt 3:1): *R*_f_ = 0.20 (major isomer) and 0.24 (minor isomer); ^1^H NMR (from diastereomeric mixture, CDCl_3_, 500 MHz): **(2** ***S*** **,1** ***S*** **)-1d** (major isomer): *δ* 1.28 (s, 9H, C(C*H*_3_)_3_), 2.33 (bs, 1H, N*H*), 2.85 (dd, ^2^ *J* = 13.5, ^3^ *J* = 8.0, 1H, C*H*_2_), 3.03 (dd, ^2^ *J* = 13.5, ^3^ *J* = 6.0, 1H, ${\rm C}H_{2}^{'}$), 3.36 (dd, ^3^ *J* = 8.0, ^3^ *J* = 6.0, 1H, H-2), 3.68 (s, 3H, OC*H*_3_), 4.08 (s, 1H, H-1), 6.67 (bs, 1H, CON*H*), 7.06 (m, 2H, H–Ar), 7.10 (m, 2H, H–Ar), 7.21–7.37 (m, 6H, H–Ar); **(2** ***S*** **,1** ***R*** **)-1d** (minor isomer): *δ* 1.08 (s, 9H, C(C*H*_3_)_3_), 2.68 (dd, ^2^ *J* = 13.5, ^3^ *J* = 10.0, 1H, C*H*_2_), 3.47 (dd, ^3^ *J* = 10.0, ^3^ *J* = 4.0, 1H, H-2), 3.75 (s, 3H, OC*H*_3_), 3.96 (s, 1H, H-1), 6.78 (bs, 1H, CON*H*), the remaining signals overlap with the signals of **(2** ***S*** **,1** ***S*** **)-1d**; ^13^C NMR (from diastereomeric mixture, CDCl_3_, 125 MHz): **(2** ***S*** **,1** ***S*** **)-1d** (major isomer): *δ* 28.6 (C(*C*H_3_)_3_), 39.4 (*C*H_2_), 50.8 (*C*(CH_3_)_3_), 51.9 (O*C*H_3_), 60.4 (C-2), 66.4 (C-1), 126.8 (C-4″), 127.6 (C-2′, C-6′), 128.1 (C-4′), 128.5 (C-2″, C-6″), 128.7 (C-3′, C-5′), 129.3 (C-3″, C-5″), 137.0 (C-1″), 138.4 (C-1′), 170.7 (*C*ONH), 174.1 (*C*OOCH_3_); **(2** ***S*** **,1** ***R*** **)-1d** (minor isomer): *δ* 28.4 (C(*C*H_3_)_3_), 40.2 (*C*H_2_), 50.3 (*C*(CH_3_)_3_), 52.1 (O*C*H_3_), 62.4 (C-2), 66.8 (C-1), 127.0 (C-4″), 127.2 (C-2′, C-6′), 128.1 (C-4′), 128.7 (C-2″, C-6″), 128.8 (C-3′, C-5′), 129.5 (C-3″, C-5″), 137.6 (C-1″), 139.5 (C-1′), 170.5 (*C*ONH), 174.8 (*C*OOCH_3_); HRMS (ESI+) calcd for C_22_H_28_N_2_O_3_Na: 391.1998 (M+Na)^+^ found 391.1995.

#### Methyl (2*S*,1*S*)- and (2*S*,1*R*)-2-(2-(*tert*-butylamino)-2-oxo-1-phenylethylamino)-3-phenylacetate **(2*S*,1*S*)-1e** and **(2*S*,1*R*)-1e**

From l-phenylglycine (3.05 g, 20.16 mmol), benzaldehyde (16.80 mmol, 1.71 mL) and *tert*-butyl isocyanide (2.00 mL, 16.80 mmol); FC (gradient: PE/AcOEt 5:1–1:2): yield 2.41 g (41 %) of chromatographically inseparable diastereomeric mixture (*d*_r_ = 1.5/1, ^1^H NMR). Pale-yellow wax; mp 65–71 °C; IR (KBr): 700, 733, 1223, 1454, 1516, 1678, 1740, 2872, 2930, 2966, 3333; TLC (PE/AcOEt 3:1): *R*_f_ = 0.28; ^1^H NMR (from diastereomeric mixture, CDCl_3_, 500 MHz): **(2** ***S*** **,1** ***S*** **)-1e** (major isomer): *δ* 1.35 (s, 9H, C(C*H*_3_)_3_), 2.85 (bs, 1H, N*H*), 3.69 (s, 3H, OC*H*_3_), 3.99 (s, 1H, H-1), 4.33 (s, 1H, H-2), 6.88 (bs, 1H, CON*H*), 7.23–7.38 (m, 10H, H–Ar); **(2** ***S*** **,1** ***R*** **)-1e** (minor isomer): *δ* 1.27 (s, 9H, C(C*H*_3_)_3_), 2.78 (bs, 1H, N*H*), 3.69 (s, 3H, OC*H*_3_), 4.05 (s, 1H, H-1), 4.29 (s, 1H, H-2), 6.97 (bs, 1H, CON*H*); the remaining signals overlap with the signals of **(2** ***S*** **,1** ***S*** **)-1e**; ^13^C NMR (from diastereomeric mixture, CDCl_3_, 125 MHz): **(2** ***S*** **,1** ***S*** **)-1e** (major isomer): *δ* 28.7 (C(*C*H_3_)_3_), 50.9 (*C*(CH_3_)_3_), 52.5 (O*C*H_3_), 63.6 (C-2), 65.1 (C-1), 127.5, 127.6 (C-2′, C-6′, C-2″, C-6″), 128.2, 128.5 (C-4′, C-4″), 128.9, 129.0 (C-3′, C-5′, C-3″, C-5″), 137.2, 139.1 (C-1′, C-1″), 170.5 (*C*ONH), 172.6 (*C*OOCH_3_); **(2** ***S*** **,1** ***R*** **)-1e** (minor isomer): *δ* 28.6 (C(*C*H_3_)_3_), 50.7 (*C*(CH_3_)_3_), 52.4 (O*C*H_3_), 64.1 (C-2), 66.9 (C-1), 127.3, 127.5 (C-2′, C-6′, C-2″, C-6″), 128.2, 128.4 (C-4′, C-4″), 128.9, 129.0 (C-3′, C-5′, C-3″, C-5″), 137.9, 139.0 (C-1′, C-1″), 170.6 (*C*ONH), 173.2 (*C*OOCH_3_); HRMS (ESI+) calcd for C_21_H_26_N_2_O_3_Na: 377.1841 (M+Na)^+^ found 377.1843.

#### Methyl (+/−)-2-(2-benzyl-2-(*tert*-butylamino)-2-oxo-1-phenylethylamino)-acetate ***rac*-1f**

From *N*-benzylglycine hydrochloride (4.06 g, 20.16 mmol), triethylamine (2.81 mL, 20.16 mmol) benzaldehyde (16.80 mmol, 1.71 mL) and *tert*-butyl isocyanide (2.00 mL, 16.80 mmol); FC (gradient: PE/AcOEt 10:1–3:1): yield 0.77 g (12 %). White powder; mp 87–89 °C; TLC (PE/AcOEt 3:1): *R*_f_ = 0.40; IR (KBr): 700, 741, 1204, 1454, 1512, 1680, 1742, 2872, 2928, 2964, 3327; ^1^H NMR (CDCl_3_, 500 MHz): *δ* 1.38 (s, 9H, C(C*H*_3_)_3_), 3.06 (d, ^2^ *J* = 17.5, 1H, PhC*H*_2_), 3.31 (d, ^2^ *J* = 17.5, 1H, Ph${\rm C}H_{2}^{'}$), 3.59 (s, 3H, OC*H*_3_), 3.67 (d, ^2^ *J* = 13.5, 1H, C*H*_2_), 3.85 (d, ^2^ *J* = 13.5, 1H, ${\rm C}H_{2}^{'}$), 4.43 (s, 1H, H-1), 7.26–7.39 (m, 10H, H–Ar), 7.60 (bs, 1H, CON*H*); ^13^C NMR (CDCl_3_, 125 MHz): *δ* 28.7 (C(*C*H_3_)_3_), 50.9 (*C*(CH_3_)_3_), 51.5 (O*C*H_3_), 51.6 (Ph*C*H_2_), 56.9 (C*H*_2_), 71.1 (C-1), 127.6, 128.1 (C-4′, C-4″), 128.5, 128.6 (C-2′, C-6′, C-2″, C-6″), 128.9, 129.6 (C-3′, C-5′, C-3″, C-5″), 135.6, 137.8 (C-1′, C-1″), 170.5 (*C*ONH), 172.1 (*C*OOCH_3_); HRMS (ESI+) calcd for C_22_H_28_N_2_O_3_Na: 391.1998 (M+Na)^+^ found 391.1985.

### Synthesis of compounds **2** by BF_3_·2CH_3_COOH mediated N-de*tert*butylation

The appropriate Ugi product **1** was dissolved in BF_3_·2CH_3_COOH (~36 % BF_3_ basis, 3 mL/1 mmol of substrate), and stirred at 45–55 °C until full conversion of the starting material is observed by TLC (typically for 4–6 h). The solution was cooled, poured onto excess of crushed ice and made alkaline with 25 % aqueous solution of ammonia. The resulting mixture was extracted with DCM (3 × 50 mL). The combined organic phase was washed with water (30 mL), brine (30 mL), dried over anhydrous MgSO_4_, filtered and concentrated under reduced pressure. The residue was purified by FC.

#### Methyl (2*S*,1*S*)- and (2*S*,1*S*)-2-(2-amino-2-oxo-1-phenylethylamino)-3-methylbutanoate **(2*S*,1*S*)-2a** and **(2*S*,1*R*)-2a**

From diastereomeric mixture of **(2** ***S*** **,1** ***S*** **)-1a** and **(2** ***S*** **,1** ***R*** **)-1a** (3.98 g, 12.43 mmol) and BF_3_·2CH_3_COOH (37 mL); FC (gradient: PE/AcOEt 2:1–0:1): yield 2.31 g (70 %): 1.95 g (59 %) of **(2** ***S*** **,1** ***S*** **)-2a**, 0.19 g (6 %) of **(2** ***S*** **,1** ***R*** **)-2a** and 0.17 g (5 %) of diastereomeric mixture. **(2** ***S*** **,1** ***S*** **)-2a**: colorless oil; [*α*]_D_ = −133.5 (*c* 0.977, CHCl_3_); IR (KBr): 702, 759, 1152, 1205, 1456, 1682, 1732, 2874, 2960, 3196, 3332, 3445; TLC (AcOEt): *R*_f_ = 0.54; ^1^H NMR (CDCl_3_, 500 MHz): *δ* 0.89 (d, ^3^ *J* = 7.0, 3H, C*H*_3_), 0.93 (d, ^3^ *J* = 7.0, 3H, ${\rm C}H_{3}^{'}$), 1.96 (m, ^3^ *J* = 7.0, 1H, C*H*), 2.22 (bs, 1H, N*H*), 2.87 (bs, 1H, H-2), 3.72 (s, 3H, OC*H*_3_), 4.19 (s, 1H, H-1), 5.80 (bs, 1H, CON*H*), 6.23 (bs, 1H, CON*H′*), 7.30–7.40 (m, 5H, H–Ar); ^13^C NMR (CDCl_3_, 125 MHz): *δ* 18.4 (*C*H_3_), 19.3 ($C{\text{H}}_{3}^{'}$), 31.4 (*C*H), 52.6 (O*C*H_3_), 64.2 (C-2), 65.6 (C-1), 128.1 (C-2′, C-6′), 128.5 (C-4′), 128.9 (C-3′, C-5′), 138.1 (C-1′), 174.3 (*C*ONH), 174.8 (*C*OOCH_3_); HRMS (ESI) calcd for C_14_H_20_N_2_O_3_Na: 287.1372 (M+Na)^+^ found 287.1396. **(2** ***S*** **,1** ***R*** **)-2a**: white powder; mp 107–109 °C; [*α*]_D_ = −5.2 (*c* 0.975, CHCl_3_); IR (KBr): 698, 758, 1150, 1202, 1456, 1685, 1733, 2874, 2960, 3196, 3331, 3443; TLC (AcOEt): *R*_f_ = 0.58; ^1^H NMR (CDCl_3_, 500 MHz): *δ* 0.96 (d, ^3^ *J* = 7.0, 3H, C*H*_3_), 1.03 (d, ^3^ *J* = 7.0, 3H, ${\rm C}H_{3}^{'}$), 2.02 (m, ^3^ *J* = 7.0, 1H, *C*H), 2.18 (bs, 1H, N*H*), 3.17 (bs, 1H, H-2), 3.72 (s, 3H, OC*H*_3_), 4.06 (s, 1H, H-1), 5.93 (bs, 1H, CON*H*), 7.22 (bs, 1H, CON*H′*), 7.28–7.44 (m, 5H, H–Ar); ^13^C NMR (CDCl_3_, 125 MHz): *δ* 18.2 (*C*H_3_), 19.6 ($C{\text{H}}_{3}^{'}$), 31.6 (*C*H), 51.8 (O*C*H_3_), 66.2 (C-1), 66.7 (C-2), 127.3 (C-2′, C-6′), 128.4 (C-4′), 128.9 (C-3′, C-5′), 138.8 (C-1′), 174.8 (*C*ONH), 174.9 (*C*OOCH_3_); HRMS (ESI) calcd for C_14_H_20_N_2_O_3_Na: 287.1372 (M+Na)^+^ found 287.1359.

#### Methyl (2*S*,1*R*)- and (2*S*,1*S*)-2-(2-amino-2-oxo-1-phenylethylamino)-4-methylpentanoate **(2*S*,1*S*)-2b** and **(2*S*,1*R*)-2b**

From diastereomeric mixture of **(2** ***S*** **,1** ***S*** **)-1b** and **(2** ***S*** **,1** ***R*** **)-1b** (3.11 g, 9.31 mmol) and BF_3_·2CH_3_COOH (28 mL); FC (gradient: PE/AcOEt 2:1–0:1): yield 1.43 g (55 %): 1.03 g (40 %) of **(2** ***S*** **,1** ***S*** **)-2b**, 0.08 g (3 %) of **(2** ***S*** **,1** ***R*** **)-2b** and 0.32 g (12 %) of diastereomeric mixture. **(2** ***S*** **,1** ***S*** **)-2b**: yellow wax; mp 65–72 °C; [*α*]_D_ = −132.9 (*c* 1.107, CHCl_3_); IR (KBr): 702, 739, 1155, 1202, 1271, 1367, 1454, 1676, 1732, 2872, 2957, 3192, 3329, 3441; TLC (AcOEt): *R*_f_ = 0.51; ^1^H NMR (CDCl_3_, 500 MHz): *δ* 0.73 (d, ^3^ *J* = 6.5, 3H, C*H*_3_), 0.87 (d, ^3^ *J* = 6.5, 3H, ${\rm C}H_{3}^{'}$), 1.47 (m, 2H, C*H*_2_), 1.76 (m, ^3^ *J*_1_ = 7.5, ^3^ *J*_1_ = 6.5, 1H, C*H*), 2.44 (bs, 1H, N*H*), 3.11 (dd, ^3^ *J*_1_ = 8.5, ^3^ *J*_1_ = 6.0, 1H, H-2), 3.70 (s, 3H, OC*H*_3_), 4.24 (s, 1H, H-1), 5.93 (bs, 1H, CON*H*), 6.31 (bs, 1H, CON*H′*), 7.29–7.39 (m, 5H, H–Ar); ^13^C NMR (CDCl_3_, 125 MHz): *δ* 21.8 (*C*H_3_), 22.9 ($C{\text{H}}_{3}^{'}$), 24.7 (*C*H), 42.6 (*C*H_2_), 51.8 (O*C*H_3_), 57.0 (C-2), 65.3 (C-1), 128.0 (C-2′, C-6′), 128.5 (C-4′), 128.9 (C-3′, C-5′), 138.2 (C-1′), 174.4 (*C*ONH), 175.5 (*C*OOCH_3_); HRMS (ESI) calcd for C_15_H_22_N_2_O_3_Na: 301.1528 (M+Na)^+^ found 301.1522; **(2** ***S*** **,1** ***R*** **)-2b**: pale-yellow powder; mp 88–95 °C; [*α*]_D_ = −0.2 (*c* 1.030, CHCl_3_); IR (KBr): 702, 756, 1157, 1202, 1269, 1387, 1454, 1680, 1734, 2870, 2957, 3190, 3325, 3445; TLC (AcOEt): *R*_f_ = 0.63; ^1^H NMR (CDCl_3_, 500 MHz): *δ* 0.95 (d, ^3^ *J* = 6.5, 3H, C*H*_3_), 0.95 (d, ^3^ *J* = 6.5, 3H, ${\rm C}H_{3}^{'}$), 1.49 (m, 2H, C*H*_2_), 1.83 (m, ^3^ *J* = 6.5, 1H, C*H*), 2.25 (bs, 1H, N*H*), 3.40 (dd, ^3^ *J*_1_ = 8.0, ^3^ *J*_1_ = 6.0, 1H, H-2), 3.70 (s, 3H, OC*H*_3_), 4.10 (s, 1H, H-1), 6.08 (bs, 1H, CON*H*), 7.17 (bs, 1H, CON*H′*), 7.27–7.42 (m, 5H, H–Ar); ^13^C NMR (CDCl_3_, 125 MHz): *δ* 22.0 (*C*H_3_), 22.9 ($C{\text{H}}_{3}^{'}$), 24.9 (*C*H), 43.1 (*C*H_2_), 51.9 (O*C*H_3_), 59.0 (C-2), 66.3 (C-1), 127.2 (C-2′, C-6′), 128.3 (C-4′), 128.8 (C-3′, C-5′), 138.7 (C-1′), 175.0 (*C*ONH), 175.7 (*C*OOCH_3_); HRMS (ESI) calcd for C_15_H_22_N_2_O_3_Na: 301.1528 (M+Na)^+^ found 301.1534.

#### Methyl (2*S*,1*R*,3*S*)- and (2*S*,1*S*,3*S*)-2-(2-amino-2-oxo-1-phenylethylamino)-3-methylpentanoate **(2*S*,1*R*,3*S*)-2c** and **(2*S*,1*S*,3*S*)-2c**

From diastereomeric mixture of **(2** ***S*** **,1** ***S*** **,3** ***S*** **)-1c** and **(2** ***S*** **,1** ***R*** **,3** ***S*** **)-1c** (3.96 g, 11.85 mmol) and BF_3_·2CH_3_COOH (35 mL); FC (gradient: PE/AcOEt 2:1–0:1): yield 2.75 g (83 %): 1.92 g (58 %) of **(2** ***S*** **,1** ***S*** **,3** ***S*** **)-2c**, 0.05 g (1 %) of **(2** ***S*** **,1** ***R*** **,3** ***S*** **)-1c** and 0.78 g (24 %) of diastereomeric mixture. **(2** ***S*** **,1** ***S*** **,3** ***S*** **)-2c**: pale-yellow oil; [*α*]_D_ = −124.1 (*c* 0.085, CHCl_3_); IR (KBr): 702, 758, 1151, 1202, 1384, 1456, 1682, 1734, 2878, 2964, 3190, 3325, 3447; TLC (AcOEt): *R*_f_ = 0.55; ^1^H NMR (CDCl_3_, 500 MHz): *δ* 0.83 (t, ^3^ *J* = 7.5, 3H, CH_2_C*H*_3_), 0.85 (d, ^3^ *J* = 7.0, 3H, C*H*_3_), 1.17 (m, 1H, C*H*_2_), 1.52 (m, 1H, ${\rm C}H_{2}^{'}$), 1.71 (m, 1H, C*H*), 2.54 (bs, 1H, N*H*), 2.94 (d, ^3^ *J* = 6.0, 1H, H-2), 3.71 (s, 3H, OC*H*_3_), 4.19 (s, 1H, H-1), 5.73 (bs, 1H, CON*H′*), 6.23 (bs, 1H, CON*H*), 7.31–7.42 (m, 5H, H–Ar); ^13^C NMR (CDCl_3_, 125 MHz): *δ* 11.3, 15.6 (*C*H_3_, $C{\text{H}}_{3}^{'}$), 25.2 (*C*H_2_), 38.0 (*C*H), 51.6 (O*C*H_3_), 63.2 (C-2), 65.6 (C-1), 128.1 (C-2′, C-6′), 128.5 (C-4′), 128.9 (C-3′, C-5′), 138.1 (C-1′), 174.3 (*C*ONH), 174.8 (*C*OOCH_3_); HRMS (ESI) calcd for C_15_H_22_N_2_O_3_Na: 301.1528 (M+Na)^+^ found 301.1516; **(2** ***S*** **,1** ***R*** **,3** ***S*** **)-2c**: white wax; mp 86–89 °C; [*α*]_D_ =+6.0 (*c* 0.833, CHCl_3_); IR (KBr): 700, 756, 1150, 1202, 1267, 1381, 1456, 1680, 1732, 2878, 2964, 3194, 3331, 3443; TLC (AcOEt): *R*_f_ = 0.63; ^1^H NMR (CDCl_3_, 500 MHz): *δ* 0.91 (t, ^3^ *J* = 7.5, 3H, CH_2_C*H*_3_), 0.97 (d, ^3^ *J* = 7.0, 3H, C*H*_3_), 1.20 (m, 1H, C*H*_2_), 1.54 (m, 1H, ${\rm C}H_{2}^{'}$), 1.76 (m, 1H, C*H*), 2.22 (bs, 1H, N*H*), 3.25 (d, ^3^ *J* = 5.5, 1H, H-2), 3.71 (s, 3H, OC*H*_3_), 4.06 (s, 1H, H-1), 6.06 (bs, 1H, CON*H′*), 7.20 (bs, 1H, CON*H*), 7.28–7.42 (m, 5H, H–Ar); ^13^C NMR (CDCl_3_, 125 MHz): *δ* 11.6, 15.9 (*C*H_3_, $C{\text{H}}_{3}^{'}$), 25.2 (*C*H_2_), 38.5 (*C*H), 51.7 (O*C*H_3_), 65.3 (C-2), 66.7 (C-1), 127.3 (C-2′, C-6′), 128.3 (C-4′), 128.9 (C-3′, C-5′), 138.8 (C-1′), 174.8 (*C*ONH), 175.0 (*C*OOCH_3_); HRMS (ESI) calcd for C_15_H_22_N_2_O_3_Na: 301.1528 (M+Na)^+^ found 301.1501.

#### Methyl (2*S*,1*R*)- and (2*S*,1*S*)-2-(2-amino-2-oxo-1-phenylethylamino)-3-phenylpropanoate **(2*S*,1*R*)-2d** and **(2*S*,1*S*)-2d**

From diastereomeric mixture of **(2** ***S*** **,1** ***S*** **)-1d** and **(2** ***S*** **,1** ***R*** **)-1d** (2.34 g, 6.36 mmol) and BF_3_·2CH_3_COOH (19 mL); FC (gradient: PE/AcOEt 2:1–0:1): yield 1.32 g (67 %): 1.10 g (55 %) of **(2** ***S*** **,1** ***S*** **)-2d** and 0.22 g (12 %) of **(2** ***S*** **,1** ***R*** **)-2d**. **(2** ***S*** **,1** ***S*** **)-2d**: pale-yellow oil; [*α*]_D_ = −72.3 (*c* 0.392, CHCl_3_); IR (KBr): 702, 752, 1205, 1454, 1682, 1734, 2854, 2951, 3028, 3190, 3325, 3445; TLC (AcOEt): *R*_f_ = 0.46; ^1^H NMR (CDCl_3_, 500 MHz): *δ* 2.40 (bs, 1H, N*H*), 2.85 (dd, ^2^ *J* = 13.5, ^3^ *J* = 8.0, 1H, C*H*_2_), 3.03 (dd, ^2^ *J* = 13.5, ^3^ *J* = 6.0, 1H, ${\rm C}H_{2}^{'}$), 3.38 (bpt, ^3^ *J* = 6.0, 1H, H-2), 3.67 (s, 3H, OC*H*_3_), 4.22 (s, 1H, H-1), 5.60 (bs, 1H, CON*H*), 6.44 (bs, 1H, CON*H′*), 7.09 (m, 2H, H–Ar), 7.12 (m, 2H, H–Ar), 7.21–7.30 (m, 6H, H–Ar); ^13^C NMR (CDCl_3_, 125 MHz): *δ* 39.4 (*C*H_2_), 51.9 (O*C*H_3_), 60.1 (C-2), 65.3 (C-1), 126.8 (C-4″), 127.7 (C-2′, C-6′), 128.3 (C-4′), 128.4 (C-2″, C-6″), 128.8 (C-3′, C-5′), 129.2 (C-3″, C-5″), 136.9 (C-1″), 137.7 (C-1′), 174.1 (*C*OOCH_3_), 174.2 (*C*ONH); HRMS (ESI) calcd for C_18_H_20_N_2_O_3_Na: 335.1372 (M+Na)^+^ found 335.1363. **(2** ***S*** **,1** ***R*** **)-2d**: white powder; mp 124–127 °C; [*α*]_D_ = −37.8 (*c* 0.775, CHCl_3_); IR (KBr): 702, 739, 1209, 1452, 1693, 1734, 2951, 3030, 3188, 3335, 3429; TLC (AcOEt): *R*_f_ = 0.58; ^1^H NMR (CDCl_3_, 500 MHz): *δ* 2.21 (bs, 1H, N*H*), 2.68 (dd, ^2^ *J* = 13.5, ^3^ *J* = 10.0, 1H, C*H*_2_), 3.11 (dd, ^2^ *J* = 13.5, ^3^ *J* = 4.0, 1H, ${\rm C}H_{2}^{'}$), 3.47 (bps, ^3^ *J* = 6.0, 1H, H-2), 3.76 (s, 3H, OC*H*_3_), 4.08 (s, 1H, H-1), 5.04 (bs, 1H, CON*H*), 6.32 (bs, 1H, CON*H′*), 7.23–7.42 (m, 10H, H–Ar); ^13^C NMR (CDCl_3_, 125 MHz): *δ* 40.1 (*C*H_2_), 52.2 (O*C*H_3_), 62.3 (C-2), 66.4 (C-1), 127.0 (C-4″), 127.3 (C-2′, C-6′), 128.4 (C-4′), 128.6 (C-2″, C-6″), 128.9 (C-3′, C-5′), 129.6 (C-3″, C-5″), 137.7 (C-1″), 138.6 (C-1′), 174.5 (*C*OOCH_3_), 174.6 (*C*ONH); C_18_H_20_N_2_O_3_Na: 335.1372 (M+Na)^+^ found 335.1366.

#### Methyl (2*S*,1*S*)- and (2*S*,1*R*)-2-(2-amino-2-oxo-1-phenylethylamino)-3-phenylacetate **(2*S*,1*S*)-2e** and **(2*S*,1*R*)-2e**

From diastereomeric mixture of **(2** ***S*** **,1** ***S*** **)-1e** and **(2** ***S*** **,1** ***R*** **)-1e** (2.26 g, 6.38 mmol) and BF_3_·2CH_3_COOH (19 mL); FC (gradient: PE/AcOEt 4:1–1:2): yield 1.54 g (81 %) of diastereomeric mixture (*d*_r_ = 1.4/1, ^1^H NMR). Pale-yellow, sticky wax; mp 43–50 °C; IR (KBr): 700, 741, 1175, 1209, 1456, 1510, 1558, 1682, 1736, 2852, 2924, 3194, 3333, 3443; TLC (AcOEt): *R*_f_ = 0.51; ^1^H NMR (from diastereomeric mixture, CDCl_3_, 500 MHz): **(2** ***S*** **,1** ***S*** **)-2e** (major isomer): *δ* 2.85 (bs, 1H, N*H*), 3.69 (s, 3H, OC*H*_3_), 4.13 (s, 1H, H-1), 4.39 (s, 1H, H-2), 5.29 (bs, 1H, CON*H*), 6.83 (bs, 1H, CON*H′*), 7.28–7.40 (m, 10H, H–Ar); **(2** ***S*** **,1** ***R*** **)-2e** (minor isomer): *δ* 2.98 (bs, 1H, N*H*), 3.68 (s, 3H, OC*H*_3_), 4.33 (s, 1H, H-2), 5.29 (bs, 1H, CON*H*), 6.80 (bs, 1H, CON*H′*); the remaining signals overlap with the signals of **(2** ***S*** **,1** ***S*** **)-2e**; ^13^C NMR (from diastereomeric mixture, CDCl_3_, 125 MHz): **(2** ***S*** **,1** ***S*** **)-2e** (major isomer): *δ* 52.4 (O*C*H_3_), 63.3 (C-2), 64.3 (C-1), 127.6 (C-2′, C-6′, C-2″, C-6″), 128.5, 128.6 (C-4′, C-4″), 129.0 (C-3′, C-5′, C-3″, C-5″), 137.1, 138.3 (C-1′, C-1″), 172.6 (*C*ONH), 174.3 (*C*OOCH_3_); **(2** ***S*** **,1** ***R*** **)-2e** (minor isomer): *δ* 52.5 (O*C*H_3_), 63.5 (C-2), 65.3 (C-1), 127.4, 127.7 (C-2′, C-6′, C-2″, C-6″), 128.4, 128.5 (C-4′, C-4″), 128.9, 129.0 (C-3′, C-5′, C-3″, C-5″), 137.3, 138.1 (C-1′, C-1″), 173.0 (*C*ONH), 174.4 (*C*OOCH_3_); HRMS (ESI+) calcd for C_17_H_18_N_2_O_3_Na: 321.1215 (M+Na)^+^ found 321.1227.

#### Methyl (+/−)-2-(2-benzyl-2-amino-2-oxo-1-phenylethylamino)-acetate ***rac*-2f**

From ***rac*** **-1f** (0.59 g, 1.60 mmol) and BF_3_·2CH_3_COOH (5 mL); FC (gradient: PE/AcOEt 4:1–1:2): yield 0.40 g (80 %) of ***rac*** **-2f**. White powder; mp 147–149 °C; TLC (AcOEt): *R*_f_ = 0.63; IR (KBr): 700, 741, 1204, 1454, 1558, 1682, 1734, 2844, 2951, 3030, 3182, 3418; ^1^H NMR (CDCl_3_, 500 MHz): *δ* 3.07 (d, ^2^ *J* = 17.5, 1H, PhC*H*_2_), 3.40 (d, ^2^ *J* = 17.5, 1H, Ph${\rm C}H_{2}^{'}$), 3.61 (s, 3H, OC*H*_3_), 3.66 (d, ^2^ *J* = 13.5, 1H, C*H*_2_), 3.85 (d, ^2^ *J* = 13.5, 1H, ${\rm C}H_{2}^{'}$), 4.75 (s, 1H, H-1), 5.85 (bs, 1H, CON*H*), 7.26–7.42 (m, 10H, H–Ar), 7.63 (bs, 1H, CON*H′*); ^13^C NMR (CDCl_3_, 125 MHz): *δ* 51.7 (O*C*H_3_), 51.8 (Ph*C*H_2_), 56.8 (C*H*_2_), 69.9 (C-1), 127.7, 128.4 (C-4′, C-4″), 128.64, 128.65 (C-2′, C-6′, C-2″, C-6″), 129.0, 129.6 (C-3′, C-5′, C-3″, C-5″), 134.7, 137.5 (C-1′, C-1″), 172.3 (*C*ONH), 174.4 (*C*OOCH_3_); HRMS (ESI+) calcd for C_18_H_20_N_2_O_3_Na: 335.1360 (M+Na)^+^ found 335.1372.

### Synthesis of compounds **3** by base-induced intramolecular cyclocondensation

To a stirred solution of appropriate amidoester **2** in absolute EtOH (5 mL/1 mmol of amidoester), sodium hydroxide (1 equiv.) was added at room temperature. After dissolution of the hydroxide, the mixture was quenched with saturated aqueous solution of ammonium chloride (100 mL). The resulting cloudy solution was extracted with CH_2_Cl_2_ (3 × 30 mL). The combined organic phase was washed with water (20 mL), dried over anhydrous MgSO_4_, filtered and concentrated under reduced pressure. The residue was purified by FC.

#### (3*S*,5*R*)- and (3*S*,5*S*)-3-isopropyl-5-phenylpiperazine-2,6-dione **(3*S*,5*S*)-3a** and **(3*S*,5*R*)-3a**

From **(2** ***S*** **,1** ***S*** **)-2a** (1.86 g, 7.04 mmol) and NaOH (0.28 g, 1 equiv.); FC (gradient: PE/EtOAc 6:1–1:1): yield 1.34 g (82 %): 0.72 g (44 %) of **(3** ***S*** **,5** ***S*** **)-3a**, 0.32 g (19 %) of **(3** ***S*** **,5** ***R*** **)-3a** and 0.30 g (19 %) of diastereomeric mixture. **(3** ***S*** **,5** ***S*** **)-3a**: white powder; mp 103–105 °C; [*α*]_D_ = −152.1 (*c* 1, CHCl_3_); IR (KBr): 756, 1030, 1099, 1180, 1234, 1331, 1454, 1497, 1701, 2932, 1963, 3225; TLC (PE/AcOEt 3:1): *R*_f_ = 0.35; ^1^H NMR (CDCl_3_, 500 MHz): *δ* 0.99 (d, ^3^ *J* = 7.0, 3H, C*H*_3_), 1.09 (d, ^3^ *J* = 7.0, 3H, ${\rm C}H_{3}^{'}$), 2.18 (bs, 1H, N*H*), 2.49 (2 sp, ^3^ *J*_1_ = 6.5, ^3^ *J*_2_ = 5.0, 1H, C*H*), 3.26 (d, ^3^ *J* = 4.5, 1H, H-3), 4.90 (s, 1H, H-5), 7.32–7.46 (m, 5H, H–Ar), 8.34 (bs, 1H, CON*H*CO); ^13^C NMR (CDCl_3_, 125 MHz): *δ* 17.1 (C*H*_3_), 19.3 (C*H*_3_), 27.7 (C*H*), 58.7 (C-3), 59.8 (C-5), 127.1 (C-2′, C-6′), 128.5 (C-4′), 129.0 (C-3′, C-5′), 134.6 (C-1′), 172.3 (C-6), 173.1 (C-2); HRMS (ESI+) calcd for C_13_H_16_N_2_O_2_Na: 255.1109 (M+Na)^+^ found 255.1101. **(3** ***S*** **,5** ***R*** **)-3a**: white powder; mp 111–112 °C; [*α*]_D_ = −117.5 (*c* 1, CHCl_3_); IR (KBr): 756, 1223, 1269, 1497, 1701, 2874, 2936, 3032, 3221; TLC (PE/AcOEt 3:1): *R*_f_ = 0.29; ^1^H NMR (CDCl_3_, 500 MHz): *δ* 1.02 (d, ^3^ *J* = 7.0, 3H, C*H*_3_), 1.09 (d, ^3^ *J* = 7.0, 3H, ${\rm C}H_{3}^{'}$), 1.76 (bs, 1H, N*H*), 2.60 (2 sp, ^3^ *J*_1_ = 7.0, ^3^ *J*_2_ = 2.5, 1H, C*H*), 3.58 (d, ^3^ *J* = 2.5, 1H, H-3), 4.54 (s, 1H, H-5), 7.36–7.44 (m, 5H, H–Ar), 8.13 (bs, 1H, CON*H*CO); ^13^C NMR (CDCl_3_, 125 MHz): *δ* 16.7 (C*H*_3_), 19.3 (${\rm C}H_{3}^{'}$), 28.8 (C*H*), 64.3 (C-3), 64.3 (C-5), 128.6 (C-2′, C-6′), 128.8 (C-3′, C-5′), 128.9 (C-4′), 136.4 (C-1′), 171.6 (C-6), 172.3 (C-2); HRMS (ESI+) calcd for C_13_H_16_N_2_O_2_Na: 255.1109 (M+Na)^+^ found 255.1129.

#### (3*S*,5*S*)- and (3*S*,5*R*)-3-isobutyl-5-phenylpiperazine-2,6-dione **(3*S*,5*S*)-3b** and **(3*S*,5*R*)-3b**

From **(2** ***S*** **,1** ***S*** **)-2b** (0.92 g, 3.31 mmol) and NaOH (0.13 g, 1 equiv.); FC (gradient: PE/EtOAc 5:1–2:1): yield 0.63 g (77 %) of chromatographically inseparable diastereomeric mixture (*d*_r_ = 68/32, ^1^H NMR). Pure **(3** ***S*** **,5** ***S*** **)-3b** was obtained by fractional recrystallization form PE/Et_2_O 1:1. **(3** ***S*** **,5** ***S*** **)-3b**: white powder; mp 60–61 °C; [*α*]_D_ = −30.3 (*c* 1, CHCl_3_); IR (KBr): 756, 1242, 1384, 1454, 1701, 2870, 2955, 3090, 3225, 3321; TLC (PE/AcOEt 3:1): *R*_f_ = 0.36; ^1^H NMR (CDCl_3_, 500 MHz): *δ* 0.84 (d, ^3^ *J* = 6.5, 3H, C*H*_3_), 0.97 (d, ^3^ *J* = 6.5, 3H, ${\rm C}H_{3}^{'}$), 1.57 (m, ^2^ *J* = 13.5, ^3^ *J*_1_ = 9.5, ^3^ *J*_2_ = 4.0, 1H, C*H*_2_), 1.85 (m, 1H, ${\rm C}H_{2}^{'}$), 1.89 (m, 1H, C*H*), 2.07 (bs, 1H, NH), 3.44 (pd, ^3^ *J* = 9.5, 1H, H-3), 4.86 (s, 1H, H-5), 7.30–7.47 (m, 5H, H–Ar), 8.34 (bs, 1H, CON*H*CO); ^13^C NMR (CDCl_3_, 125 MHz): *δ* 21.1 (*C*H_3_), 23.3 ($C{\text{H}}_{3}^{'}$), 24.4 (*C*H), 38.7 (*C*H_2_), 52.1 (C-3), 59.7 (C-5), 127.2 (C-2′, C-6′), 128.5 (C-4′), 128.9 (C-3′, C-5′), 134.7 (C-1′), 172.3 (C-6), 174.3 (C-2); HRMS (ESI+) calcd for C_14_H_18_N_2_O_2_Na: 269.1266 (M+Na)^+^ found 269.1231; **(3** ***S*** **,5** ***R*** **)-3b**: ^1^H NMR (from diastereomeric mixture, CDCl_3_, 500 MHz): *δ* 0.95 (d, ^3^ *J* = 6.5, 3H, C*H*_3_), 0.98 (d, ^3^ *J* = 6.5, 3H, ${\rm C}H_{3}^{'}$), 1.61 (m, 1H, C*H*_2_), 1.87 (m, 2H, C*H*, N*H*), 2.02 (m, ^2^ *J* = 14.0, ^3^ *J*_1_ = 10.0, ^3^ *J*_2_ = 4.0, 1H, ${\rm C}H_{2}^{'}$), 3.66 (m, 1H, H-3), 4.57 (s, 1H, H-5), 8.18 (bs, 1H, CON*H*CO), the remaining signals overlap with the signals of **(3** ***S*** **,5** ***S*** **)-3b**; ^13^C NMR (from diastereomeric mixture, CDCl_3_, 125 MHz): *δ* 21.3 (*C*H_3_), 23.4 ($C{\text{H}}_{3}^{'}$), 24.5 (*C*H), 39.0 (*C*H_2_), 57.6 (C-3), 64.6 (C-5), 128.5 (C-2′, C-6′), 128.8 (C-3′, C-5′), 128.9 (C-4′), 136.3 (C-1′), 171.8 (C-6), 173.3 (C-2).

#### (3*S*,5*R*,1*S*)- and (3*S*,5*S*,1*S*)-3-*sec*-butyl-5-phenylpiperazine-2,6-dione **(3*S*,5*S*,1*S*)-3c** and **(3*S*,5*R*,1*S*)-3c**

From **(2** ***S*** **,1** ***S*** **,3** ***S*** **)-2c** (1.81 g, 6.51 mmol) and NaOH (0.26 g, 1 equiv.); FC (gradient: PE/EtOAc 6:1–2:1): yield 1.44 g (90 %): 0.75 g (47 %) of **(3** ***S*** **,5** ***S*** **,1** ***S*** **)-3c**, 0.24 g (15 %) of **(3** ***S*** **,5** ***R*** **,1** ***S*** **)-3c** and 0.45 g (28 %) of diastereomeric mixture. **(3** ***S*** **,5** ***S*** **,1** ***S*** **)-3c**: white powder; mp 66–67 °C; [*α*]_D_ = −118.2 (*c* 1, CHCl_3_); IR (KBr): 760, 1030, 1177, 1230, 1454, 1693, 2874, 2932, 2963, 3206; TLC (PE/AcOEt 3:1): *R*_f_ = 0.38; ^1^H NMR (CDCl_3_, 500 MHz): *δ* 0.94 (t, ^3^ *J* = 7.0, 3H, CH_2_C*H*_3_), 1.07 (d, ^3^ *J* = 7.0, 3H, C*H*_3_), 1.26 (m, 1H, C*H*_2_), 1.47 (m, 1H, ${\rm C}H_{2}^{'}$), 2.20 (m, 2H, C*H*, N*H*), 3.30 (d, ^3^ *J* = 4.5, 1H, H-3), 4.90 (s, 1H, H-5), 7.31–7.46 (m, 5H, H–Ar), 8.25 (bs, 1H, CON*H*CO); ^13^C NMR (CDCl_3_, 125 MHz): *δ* 12.0, 16.0 (*C*H_3_, $C{\text{H}}_{3}^{'}$), 24.6 (*C*H_2_), 34.5 (*C*H), 58.5 (C-3), 59.8 (C-5), 127.0 (C-2′, C-6′), 128.5 (C-4′), 129.0 (C-3′, C-5′), 134.5 (C-1′), 172.2 (C-6), 173.2 (C-2); HRMS (ESI+) calcd for C_14_H_18_N_2_O_2_Na: 269.1266 (M+Na)^+^ found 269.1261; **(3** ***S*** **,5** ***R*** **,1** ***S*** **)-3c**: white powder; mp 138–139 °C; [*α*]_D_ = −94.5 (*c* 1, CHCl_3_); IR (KBr): 756, 1219, 1265, 1385, 1701, 2874, 2932, 2962, 3225; TLC (PE/AcOEt 3:1): *R*_f_ = 0.30; ^1^H NMR (CDCl_3_, 500 MHz): *δ* 0.94 (t, ^3^ *J* = 7.5, 3H, CH_2_C*H*_3_), 1.08 (d, ^3^ *J* = 7.0, 3H, C*H*_3_), 1.39 (m, 1H, C*H*_2_), 1.53 (m, 1H, ${\rm C}H_{2}^{'}$), 1.76 (bs, 1H, N*H*), 2.29 (m, 1H, C*H*), 3.61 (bps, 1H, H-3), 4.52 (s, 1H, H-5), 7.36–7.42 (m, 5H, H–Ar), 8.11 (bs, 1H, CON*H*CO); ^13^C NMR (CDCl_3_, 125 MHz): *δ* 12.3, 16.2 (*C*H_3_, $C{\text{H}}_{3}^{'}$), 24.7 (*C*H_2_), 35.8 (*C*H), 64.4 (C-3), 64.4 (C-5), 128.6 (C-2′, C-6′), 128.8 (C-3′, C-5′), 128.9 (C-4′), 136.4 (C-1′), 171.6 (C-6), 172.4 (C-2); HRMS (ESI+) calcd for C_14_H_18_N_2_O_2_Na: 269.1266 (M+Na)^+^ found 269.1280.

#### (3*S*,5*R*)- and (3*S*,5*S*)-3-benzyl-5-phenylpiperazine-2,6-dione **(3*S*,5*S*)-3d** and **(3*S*,5*R*)-3d**

From **(2** ***S*** **,1** ***S*** **)-2d** (1.02 g, 3.27 mmol) and NaOH (0.13 g, 1 equiv.); FC (gradient: PE/EtOAc 6:1–2:1): yield 0.71 g (78 %): 0.44 g (48 %) of **(3** ***S*** **,5** ***S*** **)-3d**, 0.27 g (39 %) of **(3** ***S*** **,5** ***R*** **)-3d**. **(3** ***S*** **,5** ***S*** **)-3d**: white powder; mp 114–115 °C; TLC (PE/AcOEt 3:1): *R*_f_ = 0.34; [*α*]_D_ = −88.2 (*c* 1, CHCl_3_); IR (KBr): 764, 1261, 1342, 1450, 1497, 1701, 2812, 3028, 3159, 3263, 3287; ^1^H NMR (CDCl_3_, 500 MHz): *δ* 2.12 (bs, 1H, N*H*), 3.16 (dd, ^2^ *J* = 14.0, ^3^ *J* = 8.0, 1H, C*H*_2_), 3.25 (dd, ^2^ *J* = 14.0, ^3^ *J* = 4.5, 1H, ${\rm C}H_{2}^{'}$), 3.72 (dd, ^3^ *J*_1_ = 8.0, ^3^ *J*_2_ = 4.5, 1H, H-3), 4.82 (s, 1H, H-5), 7.21–7.36 (m, 10H, H–Ar), 8.27 (bs, 1H, CON*H*CO); ^13^C NMR (CDCl_3_, 125 MHz): *δ* 35.5 (*C*H_2_), 54.7 (C-3), 59.8 (C-5), 127.1 (C-2′, C-6′), 127.3 (C-4″), 128.5 (C-4′), 128.9 (C-2″, C-6″), 128.9 (C-3′, C-5′), 129.4 (C-3″, C-5″), 134.4 (C-1′), 136.3 (C-1″), 171.7 (C-6), 172.7 (C-2); HRMS (ESI+) calcd for C_17_H_16_N_2_O_2_Na: 303.1109 (M+Na)^+^ found 303.1132; **(3** ***S*** **,5** ***R*** **)-3d**: white powder; mp 98–99 °C; TLC (PE/AcOEt 3:1): *R*_f_ = 0.28; [*α*]_D_ = −184.2 (*c* 1, CHCl_3_); IR (KBr): 760, 1230, 1288, 1454, 1716, 2851, 3086, 3182; ^1^H NMR (CDCl_3_, 500 MHz): *δ* 1.89 (t, 1H, N*H*), 2.93 (dd, ^2^ *J* = 14.0, ^3^ *J* = 9.5, 1H, H-7), 3.62 (dd, ^2^ *J* = 14.0, ^3^ *J* = 2.5, 1H, H-7′), 3.86 (dd, ^3^ *J*_1_ = 8.0, ^3^ *J*_2_ = 2.5, 1H, H-3), 4.46 (s, 1H, H-5), 7.22–7.38 (m, 10H, H–Ar), 8.18 (bs, 1H, NH); ^13^C NMR (CDCl_3_, 125 MHz): *δ* 36.5 (*C*H_2_), 60.5 (C-3), 64.5 (C-5), 127.2 (C-4″), 128.5 (C-2′, C-6′), 128.7 (C-3′, C-5′), 128.8 (C-4′), 129.0 (C-2″, C-6″), 129.3 (C-3″, C-5″), 136.0 (C-1′), 136.7 (C-1″), 171.4 (C-6), 171.8 (C-2); HRMS (ESI+) calcd for C_17_H_16_N_2_O_2_Na: 303.1109 (M+Na)^+^ found 303.1115.

#### (3*S*,5*R*)- and (3*S*,5*S*)-3,5-diphenylpiperazine-2,6-dione **(3*S*,5*S*)-3e** and **(3*S*,5*R*)-3e**

From diastereomeric mixture of **(2** ***S*** **,1** ***S*** **)-2e** and **(2** ***S*** **,1** ***R*** **)-2e** (1.43 g, 4.80 mmol) and NaOH (0.19 g, 1 equiv.); FC (gradient: PE/EtOAc 6:1–2:1): yield 0.94 g (74 %): 0.52 g (41 %) of **(3** ***S*** **,5** ***S*** **)-3e**, 0.42 g (33 %) of **(3** ***S*** **,5** ***R*** **)-3e**. **(3** ***S*** **,5** ***S*** **)-3e**: white powder; mp 126–129 °C; TLC (PE/AcOEt 3:1): *R*_f_ = 0.17; [*α*]_D_ =+5.5 (*c* 0.887, CHCl_3_); IR (KBr): 700, 744, 1240, 1454, 1695, 2855, 2922, 3070, 3204, 3312; ^1^H NMR (CDCl_3_, 500 MHz): *δ* 2.48 (bs, 1H, N*H*), 4.76 (s, 2H, H-3, H-5), 7.36–7.44 (m, 10H, H–Ar), 8.22 (bs, 1H, CON*H*CO); ^13^C NMR (CDCl_3_, 125 MHz): *δ* 59.5 (C-3, C-5), 127.7 (C-2′, C-6′, C-2″, C-6″), 128.8 (C-4′, C-4″), 129.1 (C-3′, C-5′, C-3″, C-5″), 135.2 (C-1′, C-1″), 171.5 (C-2, C-6); HRMS (ESI−) calcd for C_16_H_13_N_2_O_2_ 265.0977 (M−H)^−^ found 265.0982. **(3** ***S*** **,5** ***R*** **)-3e**: white powder; mp 172–174 °C; TLC (PE/AcOEt 3:1): *R*_f_ = 0.10; [*α*]_D_ = 0 (*c* 0.733, CHCl_3_); IR (KBr): 698, 737, 1219, 1263, 1454, 1709, 3034, 3065, 3103, 3223, 3317; ^1^H NMR (CDCl_3_, 500 MHz): *δ* 2.22 (bs, 1H, N*H*), 4.75 (s, 2H, H-3, H-5), 7.35–7.44 (m, 6H, H–Ar), 7.45–7.49 (m, 4H, H–Ar), 8.08 (bs, 1H, CON*H*CO); ^13^C NMR (CDCl_3_, 125 MHz): *δ* 65.1 (C-3, C-5), 128.7 (C-2′, C-6′, C-2″, C-6″), 128.8 (C-3′, C-5′, C-3″, C-5″), 129.0 (C-4′, C-4″), 135.9 (C-1′, C-1″), 171.2 (C-2, C-6); HRMS (ESI−) calcd for C_16_H_13_N_2_O_2_ 265.0977 (M−H)^−^ found 265.0976.

#### (+/−)-4-Benzyl-3-phenylpiperazine-2,6-dione ***rac*-3f**

From ***rac*** **-2f** (0.32 g, 1.03 mmol) and NaOH (0.04 g, 1 equiv.); FC (gradient: PE/EtOAc 3:1–1:1): yield 0.28 g (98 %): white powder; mp 156–169 °C; TLC (PE/AcOEt 3:1): *R*_f_ = 0.22; IR (KBr): 698, 744, 1246, 1454, 1699, 2814, 2852, 2924, 3030, 3209; ^1^H NMR (CDCl_3_, 500 MHz): *δ* 3.30 (d, ^2^ *J* = 17.5, 1H, PhC*H*_2_), 3.57 (d, ^2^ *J* = 17.5, 1H, Ph${\rm C}H_{2}^{'}$), 3.63 (d, ^2^ *J* = 13.5, 1H, H-3), 3.83 (d, ^2^ *J* = 13.5, 1H, H′-3), 4.50 (s, 1H, H-5), 7.23–7.39 (m, 6H, H–Ar), 7.41 (m, 4H, H–Ar), 8.24 (bs, 1H, CON*H*CO); ^13^C NMR (CDCl_3_, 125 MHz): *δ* 51.3 (Ph*C*H_2_), 58.7 (C-3), 67.1 (C-5), 128.1, 128.8 (C-4′, C-4″), 128.1, 128.8 (C-2′, C-6′, C-2″, C-6″), 128.9, 129.0 (C-3′, C-5′, C-3″, C-5″), 134.0, 136.2 (C-1′, C-1″), 170.1 (C-6), 171.0 (C-2); HRMS (ESI−) calcd for C_17_H_15_N_2_O_2_ 279.1133 (M−H)^−^ found 279.1126.

### Pharmacological evaluation

The compounds obtained have been submitted for in vivo evaluation in the ASP of NINDS, Bethesda, USA (White *et al.*, 2002). The experiments were performed in male albino Carworth Farms No. 1 mice (weighing 18–25 g). The animals had free access to feed and water except during the actual testing period. Housing, handling, and feeding were all in accordance with recommendations contained in the “Guide for the Care and Use of Laboratory Animals.” The test compounds were dissolved or suspended in 0.5 % (v/v) aqueous solution of methylcellulose.

### The MES test

In the MES test, an electrical stimulus of 0.2 s in duration (50 mA in mice) was delivered via corneal electrodes primed with an electrolyte solution containing an anesthetic agent. Mice were tested at least two different time points (15 min, 30 min, 1 h or 4 h) following intraperitoneal administration of 30, 100, and 300 mg/kg of test compound. Abolition of the hindlimb tonic extensor component indicated the test compound’s ability to inhibit MES-induced seizure spread (White *et al.*, 2002).

### The scMET seizure test

The test utilized a dose of metrazole (pentylenetetrazole, 85 mg/kg in mice). This produced clonic seizures lasting for a period of at least 5 s in 97 % (CD_97_) of animals tested. At the anticipated time of testing, the convulsant was administered subcutaneously. The test compound was administered intraperitoneally in mice and the animals were observed over a 30 min period. Mice were tested at least two different time points (15 min, 30 min, 1 h or 4 h) following intraperitoneal administration of 30, 100, and 300 mg/kg of test compound. The absence of clonic spasms indicated a compound’s ability to abolish the effect of pentylenetetrazol on seizure threshold (White *et al.*, 2002).

### The acute neurological impairment (TOX)

Neurological toxicity induced by a compound was detected in mice or rats using the standardized rotorod test (Dunham and Miya, 1957). Mice were tested at a minimum of two different time points (15 min, 30 min, 1 h or 4 h) following intraperitoneal administration of 100 mg/kg of test compound. Rats were tested at time intervals between 0.25 and 4 h following an oral or intraperitoneal dose of 30 mg/kg. Neurological impairment was demonstrated by the inability of animals to maintain equilibrium on a 6 rpm rotation rod for a given time.

### The minimal clonic seizure test (6 Hz)

The 6 Hz screen was carried out according to the protocol originally described by Brown *et al*. (1953) and more recently by Barton *et al*. (2001) and Kaminski *et al*. (2004). It is an alternative electroshock paradigm that uses low-frequency (6 Hz), long-duration (3 s) electrical stimulation. Mice were tested at time intervals between 0.25 and 4 h following intraperitoneal doses of 100 mg/kg of test compound. Corneal stimulation (0.2 ms-duration monopolar rectangular pulses at 6-Hz for 3 s) was delivered by a constant-current device. During the stimulation, mice were manually restrained and released into the observation cage immediately after the current application. The seizures manifested in “stunned” posture associated with rearing, forelimb, automatic movements and clonus, twitching of the vibrissae and Straub-tail. The duration of the seizure activity ranged from 60 to 120 s in untreated animals. At the end of the seizure, animals resumed their normal exploratory behavior. The experimental end point was protection against the seizure. The animal was considered to be protected if it resumed its normal exploratory behavior within 10 s from the stimulation (Kaminski *et al.*, 2004).
